# Determination of Spatial Chromium Contamination of the Environment around Industrial Zones

**DOI:** 10.1155/2016/7214932

**Published:** 2016-12-01

**Authors:** Dereje Homa, Ermias Haile, Alemayehu P. Washe

**Affiliations:** Department of Chemistry, Hawassa University, 05, Hawassa, Ethiopia

## Abstract

This study was conducted to determine the spatial levels of chromium contamination of water, agricultural soil, and vegetables in the leather tanning industrial areas using spectrophotometric methods. The results showed elevated accumulation of total Cr ranging from 10.85 ± 0.885 mg/L to 39.696 ± 0.326 mg/L, 16.225 ± 0.12 mg/Kg to 1581.667 ± 0.122 mg/Kg, and 1.0758 ± 0.05348 mg/Kg to 11.75 ± 0.206 mg/Kg in water, agricultural soil, and vegetable samples, respectively. The highest levels of chromium (VI) found from the speciation study were 2.23 ± 0.032 mg/Kg and 0.322 ± 0.07 mg/L in soil and water samples, respectively, which decreased with distance from the tannery. Among the vegetables, the highest load of Cr(VI) was detected in onion root (0.048 ± 0.065 mg/Kg) and the lowest (0.004 ± 0.007 mg/Kg) in fruit of green pepper. The detected levels of Cr in all of the suggested samples were above the WHO permissible limits. The variations of the levels Cr(III) and Cr(VI) contamination of the environment with distance from the tannery were statistically significant (*p* = 0.05). Similarly, significant difference in the levels of Cr among the tested vegetables was recorded. The levels increased with decreasing distance from the effluent channel.

## 1. Introduction

Chromium is an environmentally important heavy metal commonly used in various industries including tanneries, textile, chromium plating, steel production, and refractories [1]. Hazards due to chromium environmental contamination depend critically on its oxidation state and solubility [2, 3]. Chromium exhibits several oxidation states, ranging from 0 to +6, which dictate its chemical reactivity and, therefore, its environmental and biological significance. The most common oxidation states of chromium are +3 and +6 or equivalently trivalent (Cr(III)) and hexavalent (Cr(VI)) chromium [4]. The Cr(III) is the most stable form of the element because of its strong tendency to form kinetically inert hexacoordinate complexes with water, ammonia, organic acids, sulfate, halides, and urea [5], serves as an essential nutrient in plants [6], and exhibits a significant number of health benefits in animals and humans [7]. Cr(VI), on the other hand, is acidic and is the most environmentally important state of chromium. In this form, chromium is highly soluble in water and, therefore, mobile, whereas the reduced Cr(III) form is almost insoluble in water and thus immobile [8]. Previous researchers have demonstrated that Cr(VI) is stable in oxidizing environment with pH above 6.0 [9]. Under conditions of pH 3 to 6, compounds of Cr(VI) tend to reduce to more thermodynamically stable Cr(OH)_3_ [10]. All Cr(VI) compounds are strong oxidizing agents, corrosive to flesh, and considered toxic and potentially carcinogenic [11]. Intake of large amounts of Cr(VI) can cause kidney and liver damage and skin contact is known to lead to skin ulcers.

Of the various sources of Cr in the environment, tanning industries play a major role. One of the major emerging problems of the tanning industry is the disposal of chromium-contaminated sludge [12, 13]. Tannery waste contains a complex mixture of both organic and inorganic pollutants resulting from such operations as cleaning, fleshing, splitting, tanning, shaving, and buffing of animal residues [13, 14]. Chromium compounds are ubiquitous among the inorganic pollutants as a result of its use as tanning agents in the form of Cr_2_(SO_4_)_3_ and the lack of proper wastewater treatment strategy [15]. From the total Cr used in the tanning process, only 60 to 70% is utilized and the remaining 30 to 40% is released into the environment in the tannery effluent [16]. This inefficient use of chromium results in wastewater containing as high as 1500–3000 ppm (parts per million) and 500–1000 ppm of chromium by the conventional and the present day high-exhaust chrome tanning methods, respectively [17]. A wide range of physicochemical and biological methods or combination of both [18] is available for the removal of Cr from effluents but often does not completely remove the contaminants [19]. The conventional wastewater treatment modules rely on the fact that Cr salts precipitate with NaOH followed by the dissolution of Cr(OH)_3_ in sulfuric acid. However, the quality of the recovered solution is not always optimal due to the presence of the toxic state of the metal, lipidic substances, and other impurities [20]. New techniques for improving the recycling of chromium to reduce its impacts to the environment are available, but these technologies are limited to developed countries due to the high operational cost and some of them are complicated for management [21]. Although Cr(III) is the most expected form in the tannery effluents, an increase in the hexavalent form can occur as a result of redox reactions occurring in the sludge, for instance, in water by manganese oxides and in soils by mobile ligands such as citric acid, diethylene triamine pentaacetic acid (DTPA), and fulvic acid mediated oxidation [22]. The amount of chromium at any particular time depends on the intensity of industrial processes, proximity to the sources, the amount of chromium released, and meteorological factors [23]. Chromium from sources releasing the element in lager particles (particle diameter varies witin 0.2–50 mm) is deposited locally and can migrate through individual, particular environmental media. The distance covered by a deposited metal in the environment depends on meteorological factors, topography, and vegetation [1]. Transport within the terrestrial and water systems is greatly affected by chemical speciation: chemical forms of chromium and their affinity to chemical and photochemical redox transformations, precipitation/dissolution, and adsorption/desorption process, for example, occurring in individual compartments of the biogeochemical cycle of chromium [4]. Redox conversion of Cr(III) to Cr(VI) can increase Cr(VI) dislocation from the soil into the water systems [23].

In the current study, the speciation of Cr in soil, water, and vegetable samples collected at different places in the vicinity of Ethiopia Tannery Share Company was carried out and the implications were investigated. Several analytical techniques including inductively coupled plasma-mass spectrometry, inductively coupled plasma-atomic emission spectrometry, electrochemical analysis, spectrophotometry, neutron activation analysis, and atomic absorption spectrophotometry (AAS) are available for the determination and speciation of Cr(III) and Cr(VI) either in off-line or on-line methods [24]. In the off-line methods, separation and preconcentration of a particular chromium species are carried out using sample pretreatment technique such as color complex formation, soluble membrane filter techniques, chromatographic methods, coprecipitation, ion-exchange, and solvent extraction before the sample introduction in to the detection instrument. In the on-line methods, the separation system is coupled with the detection system which is difficult to simulate at laboratory conditions. In this study, off-line procedures are employed in the spectrophotometric determination and speciation of Cr(III) and Cr(VI). Complexation of Cr(VI) with 1,5-diphenylcarbazide and determination of its concentration spectrophotometrically were used as a quicker and easier method [25]. Thus, AAS was used for the determination of total Cr and UV-VIS for Cr(VI). Speciation was carried out based on the difference of results from the two methods.

## 2. Materials and Methods

### 2.1. Apparatus and Chemicals

Atomic absorption spectrophotometer (Buck Scientific, Model 210 VGP AAS, USA) equipped with deuterium background corrector and air-acetylene flame atomizer was used for determination of total Cr in the environmental samples. Spectrophotometer (model UNICAM UV-300, England) was used for determination of Cr(VI). All the reagents including 1,5-diphenylcarbazide, H_2_O_2_, HNO_3_, Cr_2_(SO_4_)_3_, and K_2_Cr_2_O_7_ were of analytical grade and used as received from the supplier (Aldrich, ACS Reagent, Germany). Serial dilutions of standard solutions for AAS analysis were prepared from standard stock solutions (Buck Scientific Calibration Standards, USA) containing 1000 mg/L of total Cr. Milli-Q water was used for all solution preparations.

### 2.2. Study Design and Description of the Study Area

The research was conducted at places in the vicinity of the Ethiopia Tannery Share Company (ETSC), in Ejersa area which is located 85 km Southeast of Addis Ababa with a grid reference of 8°27.154′ latitude and 39°03.894 longitudes. This area is characterized by a semiarid climate having an altitude of 1630 m, an average annual rainfall of 800 mm, and minimum and maximum temperature of 17.5°C and 26°C, respectively. The target area selected for collection of effluent, soil, and vegetables samples is within three-kilometer radius of effluent discharge site. As the Koka Lake (destination of the tannery effluent) is 2.5 km far away from the outlet of tannery effluent, the effluent, soil, and vegetables sampling areas were selected at the downstream section of the effluent drainage canal.

### 2.3. Sample Collection

Sampling sites were chosen based on proximity to expected anthropogenic emission sources. Additional sites away from the potential emission sources were selected for control samples. Wastewater, soil, and vegetables samples were taken at the selected sampling sites in the vicinity of the Ethiopia Tannery Share Company and 15 km farther for control samples. The sampling took place from 5 to 9 March, 2016.

Surface water samples were collected with plastic containers from five representative sampling points at 200 m with 2 hr interval from the effluent outlet to the entry point at the Lake Koka. The collected effluent samples were preserved, stored in icebox and transported to laboratory, and stored at 4°C until further analysis.

The vegetables (cabbage, green pepper, tomatoes, and onions) samples were collected at tannery effluent irrigated cultivation sites. Two groups of vegetable samples were collected. The first group consisted of 16 samples (four samples of each vegetable at different sampling sites) collected at tannery effluent irrigated cultivation sites and the second group consisted of 16 samples (four samples of each vegetable at different sampling sites) collected at cultivation sites that use normal (no tannery effluent) river water at remote areas (15 km out of tannery factory) as control samples. Each sample, from a sampling site, was taken from 10 m distance interval.

Soil samples at the surface level (0–20 cm in depth) were collected at the spot during vegetables sampling by using wood shovels and were brought to the laboratory in polyethylene plastic bags for analysis. Composite soil samples were collected from each sampling point, and each sampling position had a dimension of 20 cm width by 20 cm depth. From two sampling sites, a total of eight soil samples (four around tannery and four out of tannery area as control) were collected.

### 2.4. Preparation of Samples for Analysis

Digestion of wastewater and soil samples for total Cr determination using AAS was performed as previously described [15]. The preparation of vegetable samples for AAS analysis was also performed as previously reported but with slight modifications [26]. The vegetable samples were weighed to determine the fresh weight and dried in an oven at 80°C for 24 hours to determine their dry weight. The dried samples were ground into fine powder using pestle and mortar and passed through 1 mm sieve. Finally, 1.0 g of each of the vegetable samples was transferred into an acid-washed porcelain crucible and placed in a muffle furnace for four hours at 500°C. The crucibles were removed from the furnace and cooled. 10 mL of 6 M HCl was added, covered, and heated on a steam bath for 15 minutes. Another 1 mL of HNO_3_ was added and evaporated to dryness by continuous heating for one hour to completely digest organic compounds. Finally, 5 mL of 6 M HCl and 10 mL of deionized water were added and the mixture was heated on a steam bath to complete dissolution. The mixture was cooled and filtered through a Whatman No. 41 filter paper into a 50 mL volumetric flask and made up to mark with distilled water. For UV-Vis analysis, a 0.3 g sample of ground vegetable was weighed into a glass beaker and 25.0 mL of 0.1 M Na_2_CO_3_ was added and the mixture was boiled on a hot plate for 15 min. After filtration through Whatman No. 45 filter paper, the precipitates were washed several times with 0.1 M Na_2_CO_3_. The final volumes of the sample solutions were diluted to 25.0 mL with distilled water prior to analysis by the 1,5-diphenylcarbazide method at 540 nm. The same procedure was also applied to 1 g of soil samples. For the determination of Cr(VI) in wastewater, the wastewater samples were filtered through a Whatman No. 45 filter paper. An aliquot of 50 mL was taken from each sample and then acidified with 5 mL of 0.2 M sulfuric acid up to a mark of 50 mL. The samples were analyzed for chromium (VI) by the a UV-VIS spectrophotometer method using 1,5-diphenylcarbazide reagent (0.25 g 1,5-diphenylcarbazide in 50 mL of acetone) as complexing agent, which reacts with Cr(VI), forming a colored complex that absorbs at 540 nm.

### 2.5. Statistical Analysis

Statistical significance of the differences in the levels of Cr detected in samples of different types (soil and water, different vegetables) or sites (with reference to tannery effluent outlet point) were evaluated using one-way ANOVA at *p* = 0.05. All statistical analyses were performed using SAS Version 9.1.

## 3. Results and Discussion

### 3.1. pH Characterization of Effluent Water

**Figure 1:**
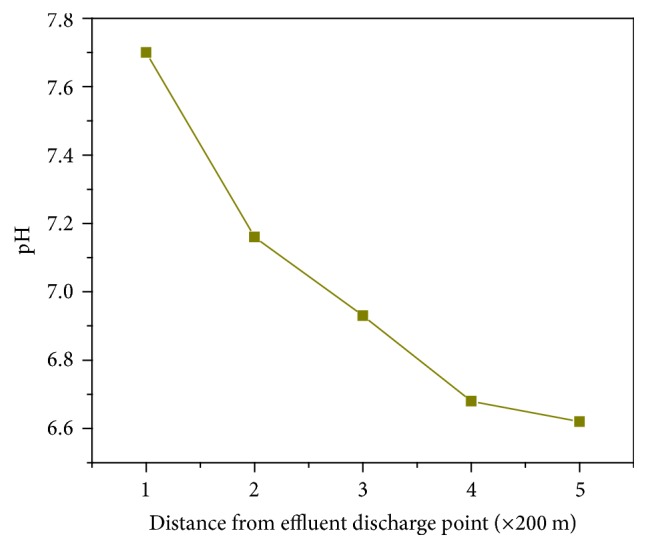
pH of water samples with distance from the source (effluent outlet).

Speciation of Cr in the environment depends,* inter alia*, on pH of the sample. Therefore, the pH of effluent water was measured prior to spectrophotometric speciation of Cr. Values of pH in the water samples taken from different points from the tannery outlet are presented in Figure 1. Generally, the pH level showed a slight variation with distance from the source which gets more acidic as distance increases. The increase in pH with distance might be due to exposure of the water to the atmosphere which can lead to dissolution of SO_2_ in the atmosphere. The pH values did not fall below 6 which means the dominant form of Cr in the environment should be the trivalent form.

### 3.2. Levels of Cr in Tannery Effluent

**Figure 2:**
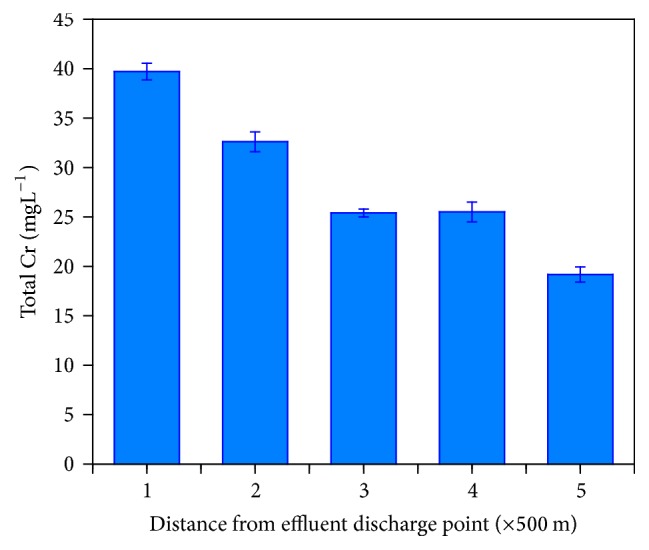
Mean values and standard deviations of total Cr concentrations recorded in wastewater samples collected at different distances from the tannery effluent discharge point.

The wastewater discharged from the Ethiopia Tannery Share Company is used for irrigation and fishing purposes by the rural community living in the vicinity of the wastewater channel downstream. Therefore, it was important to determine the level of Cr species in the tannery wastewater to comment on its suitability and/or predict the threats from its presence. Thus, the wastewater was analyzed for its total Cr concentration and speciation was carried out using the UV-Vis method. Total concentrations of Cr in wastewater samples taken at different distances along the wastewater channel were presented in Figure 2. The results indicated a decrease in the total concentration along distance from the effluent discharge point with maximum of 39.696 ± 0.326 mg/L close to the discharge point and minimum of 19.126 ± 0.864 mg/L at farther (downstream) distance from the discharge point. Moreover, the decreasing concentration of both forms of Cr along the wastewater channel was recorded which indicated the dependence of the level of environmental contamination on the intensity of industrial processes and proximity to the sources [4]. Because as the wastewater flows farther from the discharge point, adsorption of the metal within the walls of the wastewater channel soil can occur. Besides, since the Cr in the wastewater can exist either in particulate form or associated with particulates or in solution, precipitation of the particulates can result in a decrease in the Cr concentration downstream [3]. The concentrations of Cr recorded downstream (near the final discharge point: 15 km from the tannery) were also significantly higher as compared to the 1998 WHO standards for the limits of Cr in effluent discharges (Geneva) which is 1 mgL^−1^. The high value of Cr detected in the water samples above is attributed to the fact that Cr is commonly used as tanning agent and the low efficiency of the current wastewater treatment process employed by the tannery. The above findings indicated the unfortunate use of the tannery wastewater for irrigation as well as fishing might lead to health problems and other environmental implications in humans and animals.

### 3.3. Levels of Cr in Downstream Agricultural Soil

**Figure 3:**
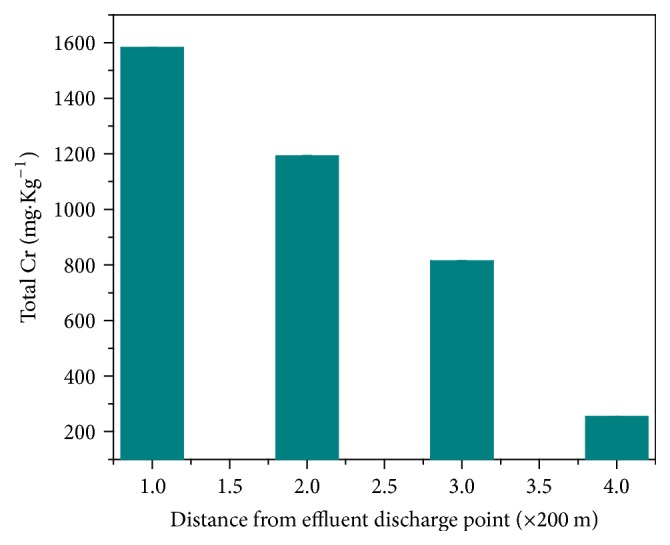
Mean values and standard deviations of total Cr concentrations recorded in soil samples collected 10 m away from the wastewater channel and at different distances from the tannery effluent discharge point.

The elevated level of Cr detected in the wastewater that decreases with distance from the effluent source indicated the possible absorption of some forms of Cr into soils. This can lead to an increase in local chromium concentration in soils. Therefore, knowledge of the level of chromium in soils in the tannery waste catchment area is important to comment on the agricultural and environmental suitability of the soils and predict the potential hazards due to the Cr presence. The average concentrations of total Cr in the agricultural soil samples collected 10 m away from the wastewater channel and at different distances from the tannery effluent discharge point were presented in Figure 3. The maximum average concentration was 1581.667 ± 0.122 mg/Kg corresponding to a sample collected at closest distance (200 m) to the effluent discharge point and the lowest concentration was 16.225 ± 0.12 mg/kg which corresponded to sample at 1 km farther from the effluent discharge point. The levels of Cr recorded in the agricultural soil samples collected up to 1 km distance farther from the tannery effluent were higher than the mean concentration of total Cr recorded in control agricultural soil collected from remote (20 km) areas. The elevated concentration of Cr in the agricultural soil in the vicinity of tanning industry might thus be attributed to the disposal of Cr contaminated sludge to the environment by the industry. All the recorded values were above the values given in the National Environmental Quality Standards (NEQS) of soil which is 20 mg/kg. Interestingly, the levels of Cr in soil samples are higher than that of water samples as expected from the continuous accumulation of the contaminant in the soil. ANOVA also showed that mean concentrations of Cr in soil and water samples were statistically significant (*p* = 0.05). In addition, the mean concentrations of soil as well as water samples varied significantly with distances from the effluent source. In addition to this, the levels of Cr in the agricultural soil in the vicinity of the tannery were significantly different from the value recorded with control soil samples which is collected from the remote areas of the tannery. The wide distribution of the contaminant along the agricultural soil might be partly due to the migration and partly because of the use of the wastewater for irrigation purpose. The above findings point to the importance of demarcation of agricultural areas with reference to the industrial areas to ensure good practice and welfare of human beings.

### 3.4. Speciation of Cr(III) and Cr(VI) in Downstream Effluent and Soil

The extent of environmental impact of Cr depends,* inter alia*, on its mobility which in turn depends on its speciation [5]. The trivalent and hexavalent forms of Cr have different mobility and environmental impact. Thus it was important to conduct speciation of the different forms of the element in the investigated environmental samples. The results of speciation study were summarized in Table 1 where it can be seen that the trivalent form is the dominant form. Although the trivalent form is the most expected form in the tannery effluents, the incidence of the hexavalent form recorded in water (max: 0.322 ± 0.07 mg/L and min: 0.12 ± 0.014 mg/L) might be attributed to redox reactions occurring in the sludge mediated by other inorganic and organic components [22]. The average concentration of chromium (VI) in soil samples ranged from 2.23 ± 0.032 mg/kg to 1.17 ± 0.02 mg/Kg with the extreme values corresponding to 200 m and 800 m sampling sites. Interestingly, concentration of Cr(VI) detected in soil samples was higher than that of water samples. This indicated accumulation of Cr(VI) in the soil samples over years of exposure to the tannery effluent. The results also showed the fact that the extent of environmental contamination or impact of both forms of the element depends on the intensity of production and proximity to the tanning industry [4]. Thus the unfortunate use of the tannery effluents for irrigation by the rural population living in the vicinity of the wastewater channel means their exposure to the potential hazards of the hexavalent Cr including its corrosive effect to flesh, toxicity, and carcinogenicity [11]. Children are more exposed to these effects as they used play with soils and mud while guarding cattle in the contaminated areas.

### 3.5. Levels of Cr in Downstream Effluent Irrigated Vegetables

**Figure 4:**
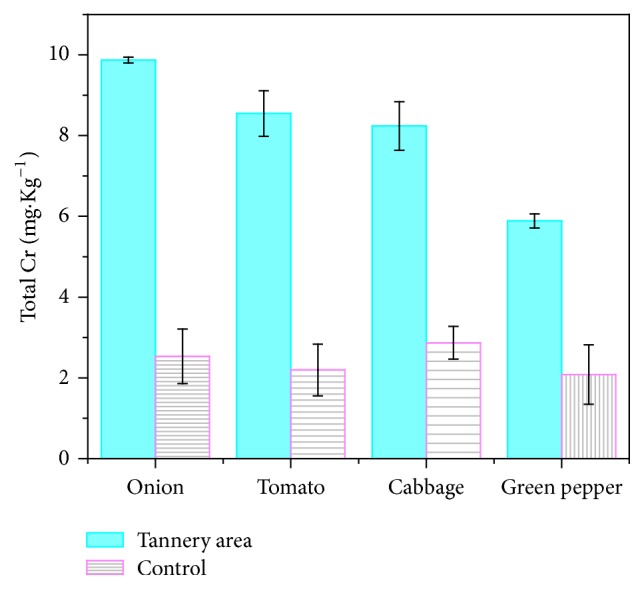
Mean values and standard deviations of total Cr concentrations recorded in tannery effluent irrigated vegetable samples cultivated around (1 km radius) tannery location.

The tannery effluent irrigated cultivation of vegetables might lead to appreciable concentration of the metal accumulated in their tissue. This not only can have impact on the productivity but also make the vegetables unsuitable for consumption. When we know that the wastewater that is used for irrigation of vegetables contains elevated concentration of Cr, we should be curious about its level in the plants. Indeed, the level of Cr that can be accumulated in plant tissues not only depends on its presence in the soil but varies among plants. In the current study, four major vegetables grown in the study area including cabbage, onion, green pepper, and tomato were tested for the level of Cr accumulated in the edible part of the plants. The average chromium concentrations (mg/kg) found in the vegetable samples collected near the Ethiopia Tannery Share Company and control areas are presented in Figure 4. The highest level of Cr was recorded in onion root (11.75 ± 0.206 mg/Kg) and the lowest level was recorded in green pepper fruit (5.75 ± 0.18 mg/Kg). The highest and the lowest levels of Cr detected in control samples were 3.341667 ± 0.075 mg/Kg in onion root and 1.075833 ± 0.053482 mg/Kg in green pepper fruits, respectively. The levels of Cr in the four vegetables follow the increasing order: green pepper fruit, tomato fruit, cabbage, and onion root. Onions were found to contain highest load of Cr. This is consistent with the fact that plants accumulate the highest level of Cr in roots [27]. The highest level of Cr recorded in onion root (11.75 ± 0.206 mg/Kg) was also comparable to the previous report (11 mg/kg) [28]. The highest level of Cr(VI) was detected in the root of onion (0.048 ± 0.065 mg/Kg) and the lowest in fruit of green pepper (0.004 ± 0.007 mg/Kg). Other authors have reported a range of Cr load in plants irrigated with tannery wastewater [8, 29]. But in the context of 167 mg·kg^−1^ of Cr detected in the plants taken from a rural area exposed to cement factory emissions [29], the levels of Cr in the tested vegetable samples in the current study were not as highly contaminated as reported before. However, the levels of Cr detected in all the investigated vegetable samples were still above the permissible limit set by WHO (1988) which is 0.19 mg·kg^−1^.

## 4. Conclusions

The speciation of Cr(III) and Cr(VI) was successfully carried out in soil, water, and vegetables samples collected at the tanning industry area using spectrophotometric methods. The study showed that all the investigated environmental samples including agricultural soil and vegetables contain high load of Cr(III). The levels of both forms of Cr (trivalent and hexavalent) in soil and water samples were above the WHO standards and showed decreasing trend with distance from the tannery effluent discharge point. Soil samples in the vicinity of the tanning industry contained elevated load of Cr indicating the unsuitability for agricultural purposes and potential hazards to human beings and animals if appropriate clean up strategy is implemented. All the tested vegetables (cabbage, onion, tomato, and green pepper) contained above permissible levels of Cr(III) in their tissue with the highest concentration found in the edible root parts of onion. Cr(VI) is not detected in the vegetable samples probably due to lack of absorption. ANOVA showed that variations of the level of Cr among the tested vegetables were statistically significant. This confirmed that the chromium absorption from the soil by vegetables depends on the plant species with highest accumulation in roots. In the agricultural soil in the vicinity of the tannery, the levels of Cr(III) and Cr(VI) were much greater compared to control soil samples collected at remote areas. In conclusion, the discharge of Cr rich sludge to the environment and the unfortunate use for agricultural purposes by the community living in the vicinity of the effluent channel can lead to potential hazards of Cr, particularly the hexavalent form, on humans and affect the quality of the natural resources around the tanning industry. Therefore, appropriate reclamation methods are crucial to reducing Cr contamination.

## Figures and Tables

**Table 1:** Speciation of Cr(III) and Cr(VI) in water, agricultural soil, and vegetables (mg·Kg^−1^).

Samples	Cr	Cr(III)	Cr(VI)
*W*_1_	39.696 ± 0.326	39.374 ± 0.256	0.322 ± 0.07
*W*_2_	32.626 ± 0.141	32.419 ± 0.096	0.207 ± 0.045
*W*_3_	25.353 ± 0.813	25.191 ± 0.743	0.162 ± 0.07
*W*_4_	25.463 ± 0.34	25.343 ± 0.326	0.12 ± 0.014
*W*5	19.126 ± 0.864	19.126 ± 0.864	ND
*S*_200_	1581.66 ± 0.12	1579.43 ± 0.088	2.23 ± 0.032
*S*_400_	1192.08 ± 1.106	1190.08 ± 1.081	1.926 ± 0.025
*S*_600_	813.66 ± 0.49	811.84 ± 0.47	1.82 ± 0.02
*S*_800_	254.25 ± 0.22	253.08 ± 0.20	1.17 ± 0.02
Root onion	10.3415 ± 0.1315	10.2935 ± 0.0665	0.048 ± 0.065
Cabbage	8.0705 ± 0.6542	8.0285 ± 0.6372	0.042 ± 0.017
Tomato	8.6227 ± 0.1535	8.5967 ± 0.1293	0.026 ± 0.024
Green pepper	5.9455 ± 0.1028	5.9415 ± 0.0958	0.004 ± 0.007

*W*_*n*_: *W* = water sample and *n* = distance from effluent source; *S*_*m*_: *S* = soil sample and *m* = distance from the effluent source.

## References

[1] Nriagu J. O., Nriagu J. O., Nieboer E. (1988). Production and uses of chromium. *Chromium in Natural and Human Environments*.

[2] Mishra S., Singh V., Srivastava S. (1995). Studies on uptake of trivalent and hexavalent chromium by maize (*Zea mays*). *Food and Chemical Toxicology*.

[3] Shrivastava R., Upreti R. K., Seth P. K., Chaturvedi U. C. (2002). Effects of chromium on the immune system. *FEMS Immunology and Medical Microbiology*.

[4] Kotaś J., Stasicka Z. (2000). Chromium occurrence in the environment and methods of its speciation. *Environmental Pollution*.

[5] Apte A. D., Verma S., Tare V., Bose P. (2005). Oxidation of Cr(III) in tannery sludge to Cr(VI): field observations and theoretical assessment. *Journal of Hazardous Materials*.

[6] Stępniewska Z., Wolińska A. (2005). Soil dehydrogenase activity in the presence of chromium (III) and (VI). *International Agrophysics*.

[7] Anderson R. A. (2000). Chromium in the prevention and control of diabetes. *Diabetes & Metabolism*.

[8] Bini C., Maleci L., Romanin A. (2008). The chromium issue in soils of the leather tannery district in Italy. *Journal of Geochemical Exploration*.

[9] James B. R., Petura J. C., Vitale R. J., Mussoline G. R. (1997). Oxidation-reduction chemistry of chromium: relevance to the regulation and remediation of chromate-contaminated soils. *Journal of Soil Contamination*.

[10] Masscheleyn P. H., Pardue J. H., DeLaune R. D., Patrick W. H. (1992). Chromium redox chemistry in a Lower Mississippi Valley bottomland hardwood wetland. *Environmental Science and Technology*.

[11] Das A. P., Mishra S. (2008). Hexavalent chromium (VI): environment pollutant and health hazard. *Journal of Environmental Research and Development*.

[12] Mwinyihija M. (2010). *Ecotoxicological Diagnosis in the Tanning Industry*.

[13] Mwinyihija M., Meharg A., Dawson J., Strachan N. J. C., Killham K. (2006). An ecotoxicological approach to assessing the impact of tanning industry effluent on river health. *Archives of Environmental Contamination and Toxicology*.

[14] Mwinyihija M., Strachan N. J. C., Meharg A., Killham K. (2005). Biosensor based toxicity dissection of tannery and associated environmental samples. *Journal of the American Leather Chemists Association*.

[15] Reda A. H. (2015). Study on the pollution levels of trace metals from modjo tannery effluent in the surrounding river water and soil. *Science Journal of Analytical Chemistry*.

[16] Cassano A., Della Pietra L., Drioli E. (2007). Integrated membrane process for the recovery of chromium salts from tannery effluents. *Industrial & Engineering Chemistry Research*.

[17] Aravindhan R., Madhan B., Rao J. R., Nair B. U., Ramasami T. (2004). Bioaccumulation of chromium from tannery wastewater: an approach for chrome recovery and reuse. *Environmental Science and Technology*.

[18] Reemtsma T., Jekel M. (1997). Dissolved organics in tannery wastewaters and their alteration by a combined anaerobic and aerobic treatment. *Water Research*.

[19] Bouwer E., Durant N., Wilson L., Zhang W., Cunningham A. (1994). Degradation of xenobiotic compounds *in situ*: capabilities and limits. *FEMS Microbiology Reviews*.

[20] Cassano A., Molinari R., Romano M., Drioli E. (2001). Treatment of aqueous effluents of the leather industry by membrane processes: a review. *Journal of Membrane Science*.

[21] Sundar V. J., Rao J. R., Muralidharan C. (2002). Cleaner chrome tanning—emerging options. *Journal of Cleaner Production*.

[22] Stepniewska Z., Bucior K., Bennicelli R. P. (2004). The effects of MnO_2_ on sorption and oxidation of Cr(III) by soils. *Geoderma*.

[23] Bartlett R. J., Kimble J. M. (1976). Behavior of chromium in soils: oxidation. *Journal of Environmental Quality*.

[24] Jyothi N. R., Farook N. A. M., Cho M., Shim J. (2013). Analysis and speciation of chromium in environmental matrices by various analytical techniques. *Asian Journal of Chemistry*.

[25] Noroozifar M., Khorasani-Motlagh M. (2003). Specific extraction of chromium as tetrabutylammonium-chromate and spectrophotometric determination by diphenylcarbazide: speciation of chromium in effluent streams. *Analytical Sciences*.

[26] Akan J. C., Kolo B. G., Yikala B. S., Ogugbuaja V. O. (2013). Determinations of some heavy metals in vegetable samples from Biu Local Government Area, Borno State, North Eastern Nigeria. *International Journal of Environmental Monitoring and Analysis*.

[27] Bai R. S., Abraham T. E. (2001). Biosorption of Cr (VI) from aqueous solution by *Rhizopus nigricans*. *Bioresource Technology*.

[28] Wolińska A., Stępniewska Z., Włosek R. (2013). The influence of old leather tannery district on chromium contamination of soils, water and plants. *Natural Science*.

[29] Isikli B., Demir T. A., Ürer S. M., Berber A., Akar T., Kalyoncu C. (2003). Effects of chromium exposure from a cement factory. *Environmental Research*.

